# Microbiome and metabolic changes in milk in response to artemisinin supplementation in dairy cows

**DOI:** 10.1186/s13568-020-01080-w

**Published:** 2020-08-24

**Authors:** Kun Hou, Jinjin Tong, Hua Zhang, Shan Gao, Yuqin Guo, Hui Niu, Benhai Xiong, Linshu Jiang

**Affiliations:** 1 Beijing Key Laboratory for Dairy Cow Nutrition, Beijing University of Agriculture, Beijing, 102206 People’s Republic of China; 2 State Key Laboratory of Animal Nutrition, Institute of Animal Science, Chinese Academy of Agricultural Sciences, Beijing, 100193 People’s Republic of China

**Keywords:** Microbiome, Metabolomics, Artemisinin, Milk, Dairy cows

## Abstract

This study aimed to explore the effects of artemisinin (ART) on the milk microbiome and metabolites of dairy cow. A total of 12 mid-lactation Holstein dairy cows with similar parity, days in milk were randomly divided into 2 groups receiving either a total mixed ration (TMR) as the control group or this TMR and 120 g/d/head ART as the ART group. The milk samples were collected weekly to determine the contents, and end-of-trial (week 8) milk samples were used to identify microbial species and metabolite profiles by 16S rRNA sequencing and LC–MS analyses, respectively. We observed that the milk fat content significantly increased by ART treatment (*P *< 0.05). The bacterial community richness was significantly lower in the ART group (*P *< 0.05), while the diversity showed no difference (*P *> 0.05). Compared with its abundance in the control (CON) group, *Firmicutes* was significantly decreased, whereas *Proteobacteria* was significantly increased. Furthermore, in the ART group, the relative abundances of the genera *Aerococcus*, *Staphylococcus, Corynebacterium_1* and *Facklamia* were significantly lower (*P *< 0.01). Metabolomics analysis revealed that ART significantly increasing the concentrations of glycerophospholipids, glycerolipids and flavonoids compared with those in the CON group. An enrichment analysis of the different metabolites showed that ART mainly affected glycerophospholipid metabolism and the pantothenate and CoA biosynthesis pathways. These findings revealed that ART supplementation could affect the milk microbiota and metabolites, that glycerophospholipids and glycerolipids could be potential biomarkers in the milk response to ART feed in dairy cows, and that ART changes substances in milk by maintaining lipid metabolism in the mammary gland.

## Introduction

Milk serves not only as an important medium for nutrition but also as an indicator of the metabolic status of dairy cows. Insights from recent investigations of milk biosynthesis suggest that the microbiota can provide a great reference for the evaluation of milk quality (Guan et al. 2014). Members of the milk microbiota could directly affect the quality of milk, such as lactic acid bacteria, Lactobacillus, and Lactococcus (Guan et al. 2014; Vanderhaeghen et al. 2014; Ganda et al. 2017). In particular, some specific dominant bacteria play an important role in udder homeostasis, which guarantees high milk quality (Tong et al. 2019).

To a certain extent, microbial diversity and richness reflect the mammary gland health of dairy cows (Guan et al. 2014), and there is an interesting report of the udder microbiota having a lower diversity in quarters with a history of clinical mastitis than in healthy quarters (Hélène et al. 2016). Additionally, 16S rRNA sequencing technology has become an important technique to study the composition and structure of sample microbial communities (Caporaso et al. 2011). Through comparison with databases, the diversity of the milk microbiome was analyzed with high speed, high flux and high accuracy (Kuehn et al. 2013). In particular, the udder commensal mammary microbiota has recently attracted much attention to the milk microbiota differences between the colostrum and milk from healthy quarters or mastitis quarters of dairy cows (Guan et al. 2014; Hélène et al. 2016). In addition, the administration of antibiotics for mastitis treatment and antimicrobial resistance also reveal the effects of the milk microbiome response to udder defense mechanisms as determined by 16S rRNA sequencing or metagenomic investigations (Ganda et al. 2017).

With variations in the microbial community structure in milk, metabolites also change. Milk quality is regulated by different metabolic pathways, resulting in differences in milk production and nutrients such as fat, protein, and lactose. Furthermore, a complex array of bioactive molecules such as immunoglobulins, lysozyme, and lactoferrin are the major immunoregulatory components of milk (Korhonen et al. 2000). Metabolomics has received much attention because it can amplify tiny changes in gene and protein expression at the metabolite level and more fully reflect the functional level of cells than other techniques can (Mansor 2012). In addition, metabolomics is also used to quantitatively measure metabolic status in milk, including the levels of metabolic biomarkers during lactation (Sun et al. 2017a), alterations in metabolites resulting from mastitis (Tong et al. 2019), and changes in metabolic profiles resulting from antibiotic treatment (Junza et al. 2016). Nontarget metabolic techniques based on liquid chromatography/mass spectrometry (LC–MS) could identify a large number of complete metabolites with the advantages of high flux and high sensitivity (Gowda and Djukovic 2013). Therefore, LC–MS can provide a comprehensive overview of changes in milk metabolites in response to artemisinin supplementation in dairy cows.

Artemisinin (ART), a sesquiterpene lactone with a peroxy-bridge structure, has multiple actions including antibacterial, anti-inflammatory, antitumor and antiviral pharmacological effects (Shi et al. 2015). Because of its effectiveness as an anti-malarial drug, artemisinin was labeled as “the greatest hope for treating malaria” by the WHO, and Chinese scientist Tu You-you won the Nobel Prize in Physiology or Medicine in 2015 for her discoveries. Moreover, artemisinin has great potential in the poultry industry. Previous studies have shown that adding appropriate amounts of ART or its derivatives to the diet improved the performance of laying hens, increased egg weight and yolk color and had anticoccidial effects (Brisibe et al. 2008). In addition, ART can effectively alleviate declines in broiler performance caused by heat stress and reduce intestinal inflammation (Song et al. 2017). ART feeding trials have shown promising bacteriostatic activity especially with regard to its potential role in the bovine rumen which houses a complex microbiota and plays an important role in digestion. However, the application of ART in dairy farming has rarely been reported. We hypothesized that ART would affect dairy cow performance and trigger changes in milk microbiota and metabolites, providing a theoretical basis of traditional Chinese herbal medicine for animal welfare.

Our goal was to appraise the effects of ART on the performance and milk microbial diversity and metabolites of dairy cows. We aimed to explore the changes in the species composition of the milk microbial profile by using 16S rRNA sequencing and to detect the metabolites in milk by untargeted metabolic techniques. We also identified correlations between the milk microbiota and metabolites by ART supplementation.

## Materials and methods

The *Artemisia annua* extract (brown powder form) used in these experiments was purchased from Shaanxi Sciphar Natural Products Co., Ltd. (Shanxi, China). The active ingredients in the *Artemisia annua* extract were analyzed by UV spectroscopy, resulting in the following contents: ART 39%, crude ash 5%, crude fiber 27.9%, crude protein 6.3%, water 5%, ash 8.0%, polysaccharide 8.3% and volatile oil 0.5% (Additional file 1).

### Animals and experimental design

All experimental procedures were approved by the Animal Care Committee, Beijing University of Agriculture (Beijing, China). A feeding experiment was performed in a commercial dairy farm in Yanqing District, Beijing. Twelve lactating Chinese Holstein dairy cows with similar weight (590 ± 15.5 kg; *P* = 0.96), parity (3.65 ± 0.78; *P* = 0.82), days in milk (183.2 ± 16.8 d; *P* = 0.65), and milk yield (30.47 ± 3.23 kg/d; *P* = 0.88) were selected and divided into two groups. The cows were fed either a total mixed ration (TMR) as the control group (CON, n = 6) or this TMR with ART supplementation of 120 g/d/head (ART, n = 6). The ART dosage for dairy cows used in the present study was based on the dosage from an in vitro study and an in vivo study in dairy cows (Xue et al. 2004; Hou et al. 2019). The animals were housed individually in stalls bedded with sawdust, feed was available for ad libitum consumption, and free access to water was given. The ingredients and nutrient composition of the TMR were presented in Table 1. Feeding and milking occurred 3 times per day (07:00, 14:00 and 20:00). The experiment lasted for 8 weeks, with 2 weeks for feeding with the TMR and 6 weeks of treatments. Milk samples were collected weekly, and production was recorded at each milking. A total of 15 ml of composite milk from each animal, with approximately equal volumes from each lactating udder quarter, was transferred to a sterile plastic bottle (Corning Inc., Corning, NY, United States), kept on ice until transport to the laboratory and then stored at −80 °C for further analysis.

**Table 1:** Composition and nutrient levels of the basal diet

Item	Content, %
Ingredient	
Ground corn	9.58
Corn silage	46.62
Corn bran	3.70
Steam-flaked corn	4.40
Alfalfa hay	6.90
Oat grass	2.50
Soybean meal	5.00
Dried distiller grains with solubles (DDGS)	4.40
MEGALAC^a^	0.90
Extruded soybean	3.00
Barley	2.76
Wheat bran	2.76
Sodium cyclamate	2.20
Oats	1.30
Canola meal	1.17
Cottonseed meal	1.17
NaHCO_3_	0.59
Limestone	0.48
NaCl	0.27
Premix^b^	0.30
Chemical composition^c^	
Crude protein (CP)	18.4
Neutral detergent fiber (NDF)	31.1
Acid detergent fiber (ADF)	15.6
Ether extract	5.00
Ca	0.77
P	0.43
NE_L_, Mcal/kg	1.76

^a^Church and Dwight Co., Inc., Princeton, NJ, USA

^b^Formulated to provide (per kg of DM) 4,560 mg of Cu, 3,000 mg of Fe, 12,100 mg of Zn, 4,590 mg of Mn, 60 mg of Co, 200 mg of Se, 270 mg of I, 10,000 IU of vitamin E, 450,000 IU of vitamin D, 2,000,000 IU of vitamin A, and 3,000 mg of nicotinic acid

^c^Chemical composition based on chemical analysis of the total mixed ration (TMR), as described

### Milk sampling and analyses

The daily milk production was recorded during the experiment. The somatic cell count (SCC) in milk was detected by a somatic cell counting instrument (Fossomatic 5000, FOSS, Denmark). The milk fat rate, milk protein rate and milk lactose rate were determined by a milk composition analyzer (Bentley Instruments, Chaska, MN). Milk samples were collected weekly to measure the contents, and the end-of-trial (week 8) milk samples were used to identify the microbial species and metabolite profiles by 16S rRNA gene sequencing and LC–MS, respectively. All the samples were stored in liquid nitrogen for further analysis.

### 16S rRNA high-throughput sequencing analysis

Genomic DNA extraction for 12 milk samples was performed using a Power Soil DNA Isolation Kit (Qiagen, Crawley, United Kingdom) following the manufacturer’s instructions. The concentration of DNA was determined by spectrophotometry, and the purity of DNA was estimated according to the ratio of the UV absorption values of DNA at 260 nm and 280 nm (OD260/OD280). Amplicon libraries covering the V3-V4 hypervariable regions of the microbial 16S rDNA gene were amplified using the primers 341F (5′-ACTCCTACGGGRSGCAGCAG-3′) and 806R (5′-GGACTACVV GGGTATCTAATC-3′). The reactions were performed on a thermocycler (GeneAmp 9700, ABI, USA). PCR amplification products were further purified by the AxyPrep DNA Gel Extraction Kit (Axygen Biosciences, Union City, CA, USA) and then quantified using QuantiFluor™-ST (Promega, USA). Finally, high-throughput sequencing analysis of bacterial rRNA genes was performed on purified pooled samples using the Illumina HiSeq platform (Illumina, San Diego, CA, USA) for paired-end reads of 300 bp at Majorbio Bio-Pharm Technology Co. Ltd. (Shanghai, China) according to standard protocols.

### Sequence processing and analysis

Analyses were conducted with FLASH version 1.2.11 and Quantitative Insights into Microbial Ecology (QIIME) ver 1.9.1. These versions gave similar data(Tong et al. 2019). The reads were clustered as operational taxonomic units (OTUs) by scripts in USEARCH (ver 7.1) with a 97% similarity threshold(Edgar, 2013). The OTU sequences were categorized by taxa by BLAST in the Ribosomal Database Project Classifier (ver 2.2) and Silva (SSU123) 16S rRNA database. OTUs were normalized to relative abundance and bacterial composition was determined by Majorbio I-Sanger.

Within-sample diversity (alpha diversity) was assessed through bacterial community enrichment (ACE and Chao indices) and diversity (Shannon and Simpson indices) that were measured in a stochastic subset of the OTUs. Between-sample microbial diversity (beta diversity) was measured by phylogenetically-based weighted UniFrac distances (Wang et al. 2018). The predominant clades in the milk microbiome were acquired by filtering OTUs for those with relative abundance of  ≥ 1% one or more samples.

### Nucleotide sequence accession number

All raw sequences were submitted to the NCBI Sequence Read Archive (SRA: http://www.ncbi.nlm.nih.gov/Traces/sra/) under accession number SRP 254006.

### Metabolomics analysis

Milk samples were analyzed for specific components using an LC–MS platform (Thermo, Ultimate 3000 LC, Q Exactive), and sample preparation was performed as per our previously published procedure (Wang et al. 2018; Tong et al. 2019). The following steps were conducted by Majorbio Bio-Pharm Technology Co., Ltd. Analysis of metabolomics data was performed with Progenesis QI (Waters Corporation, Milford, USA) to match MS and MS/MS mass spectrum information with that in the metabolism database. The retention time (RT), m/z, observation data (samples) and peak intensity were normalized using Microsoft Excel 2017. Screened differential metabolites were characterized using the https://metlin.scripps.edu/public database, a self-built database for the Majorbio I-Sanger Cloud Platform (www.i-sanger.com) and KEGG pathway analysis (www.metaboanalyst.ca/).

### Multivariate statistical analysis

Statistical comparisons were evaluated using Student’s *t* test. A *P* value of  < 0.05 was defined as statistically significant. Hierarchical clustering was conducted using the similarity index of Bray–Curtis by the UPGMA. The strengths of correlations between metabolites and milk bacterial species were estimated using Spearman correlation coefficients and visualized by using the R language (Kolde 2015). A *P* value  < 0.05 was defined as statistically significant. The statistical analyses were performed with SPSS software version 21.0 (IBM, Armonk, NY). The alpha diversity indexes are presented as the mean ± SEM. Principal coordinate analysis (PCoA) and orthogonal partial least-squares-discriminant analysis (OPLS-DA) were performed to visualize the metabolic differences between the experimental groups after mean centering and unit variance scaling. Variables with variable importance in the projection (VIP) values exceeding 1.0 were considered relevant for group discrimination. In this study, the OPLS-DA model was validated with sevenfold permutation tests. Significant differences in metabolites between groups were assessed using Wilcoxon rank-sum tests.

The original milk composition data were analyzed by Excel 2017, and statistical comparisons were evaluated using one-way ANOVA in SPSS 21.0 was used (IBM Corp., Armonk, NY, USA). Differences were considered statistically significant when *P* < 0.05 and a trend when *P* < 0.10.

## Results

### Effects of ART on milk performance

As shown in Table 2, the milk yield tended to increase (*P *= 0.06), and the fat yield (*P *= 0.04) was increased by ART supplementation at the end of the trial. Milk lactose and protein were not different in the two treatment groups. Milk fat content was greater in the ART group than in the CON group (*P *= 0.04), and the fat/protein ratio was also increased (*P *= 0.03). In addition, the SCC tended to be decreased (*P *= 0.08) with ART supplementation compared with the CON treatment.

**Table 2:** Effect of dietary addition of ART on milk production and composition in dairy cows

Item	CON	ART	SEM	*P* value
Yield, kg/d				
Milk	30.23	31.22	0.91	0.06
ECM^1^	30.50	32.42	1.03	0.14
Lactose	1.52	1.55	0.06	0.12
Fat	1.01	1.12	0.09	0.04
Protein	0.95	0.96	0.03	0.82
Milk composition, %				
Lactose	5.03	4.97	0.04	0.16
Fat	3.31	3.58	0.18	0.04
Protein	3.15	3.07	0.22	0.41
Fat/protein ratio	1.05	1.16	0.13	0.03
SCC (× 10^4^ cells/ml)	12.77	10.35	0.32	0.08

^1^ ECM (kg/d) = 0.3246 × milk yield (kg/d) + 13.86 × fat yield (kg/d) + 7.04 × protein yield (kg/d)(Orth 1992)

### Microbiota diversity analysis

In total, 757,666 high-quality sequences for 12 samples were analyzed after the sequences passed quality control, which resulted in an average read length of 311 bp, and there was  > 99% depth coverage. This result showed that the data were reasonable and could reflect the changes in most bacterial flora. Furthermore, the rarefaction curves for most of the samples plateaued, which further confirmed the sufficiency of the data. The alpha diversity index results for the groups are shown in Table 3. According to the ACE and Chao indexes, which represent the bacterial community richness, there was a significant difference between the CON and ART groups, with lower richness in the ART group (*P *< 0.05). However, the bacterial diversity (Shannon and Simpson indexes) was similar between the two groups.

**Table 3:** Effect of ART on alpha diversity indexes of milk

Index	CON	ART	SEM	*P* value
ACE	2335.08	1956.18	105.59	0.04
Chao	2258.71	1805.37	92.79	0.02
Simpson	0.05	0.08	0.01	0.35
Shannon	4.75	4.41	0.13	0.48
Coverage	0.99	0.99	0.01	0.21

**Fig. 1:**
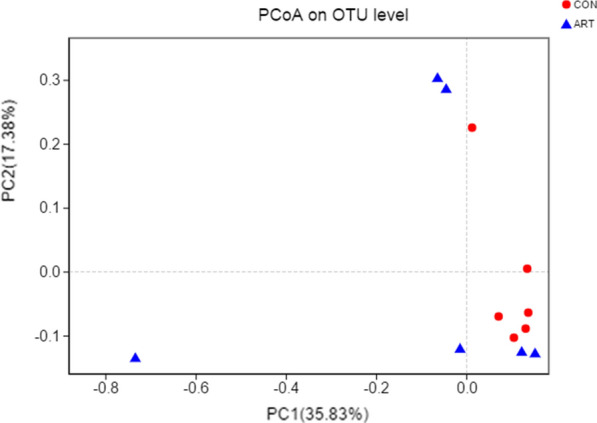
Principal coordinate analysis (PCoA) of milk microbial communities from lactating cows fed TMR supplemented with 0 (CON) and 120 g/d artemisinin (ART), n = 6

Principal coordinate analysis (PCoA) using weighted UniFrac metrics was performed to visually analyze the similarity or difference in microbial composition in the different groups (Fig. 1). The principal coordinates PC1 and PC2 accounted for 35.83% and 17.83% of the total variance, respectively. This result reflects the microflora being remarkably distinct between the two groups, and the sample points for each group were relatively close.

**Fig. 2:**
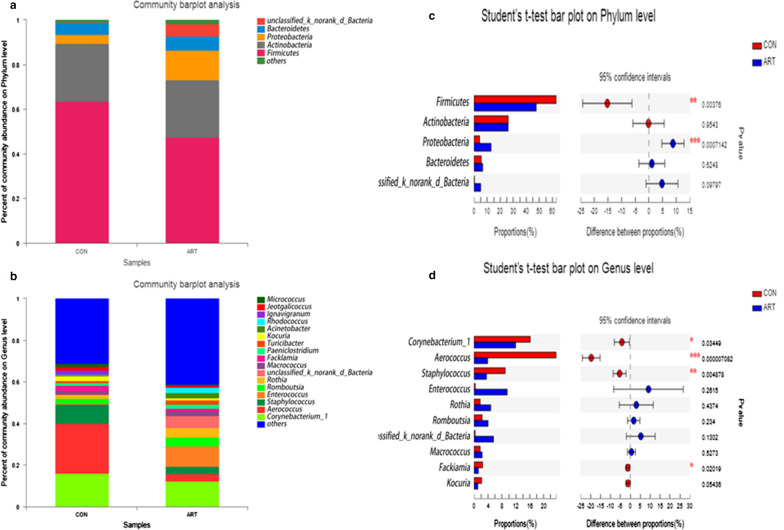
Predominant bacteria in milk samples from lactating cows fed TMR supplemented with 0 (CON) and 120 g/d artemisinin (ART), n = 6. **a** Phylum level. **b** Genus level. **c** Significant differences in bacteria at the phylum level in milk samples between the two groups. **d** Significant differences in bacteria at the genus level in milk samples between the two groups. The extended error bar plot was generated using STAMP software. Welch’s two-sided test was used, and Welch’s inverted confidence interval was 0.95

The results showed a change in the relative abundance of bacteria in milk samples, with the phylum *Firmicutes* being significantly decreased in the ART group (*P *< 0.01), whereas the relative abundance of *Proteobacteria* was higher (*P *< 0.01) in the ART group than in the CON group (Fig. 2a, c). At the genus level, the top 10 most abundant bacterial taxa were presented for the two groups (Fig. 2b, d). The ART group had significantly lower relative abundances of *Corynebacterium_1* (*P *< 0.05)*, Aerococcus* (*P *< 0.01), *Staphylococcus (P *< 0.01) and *Facklamia* (*P *< 0.05) than the CON group.

**Fig. 3:**
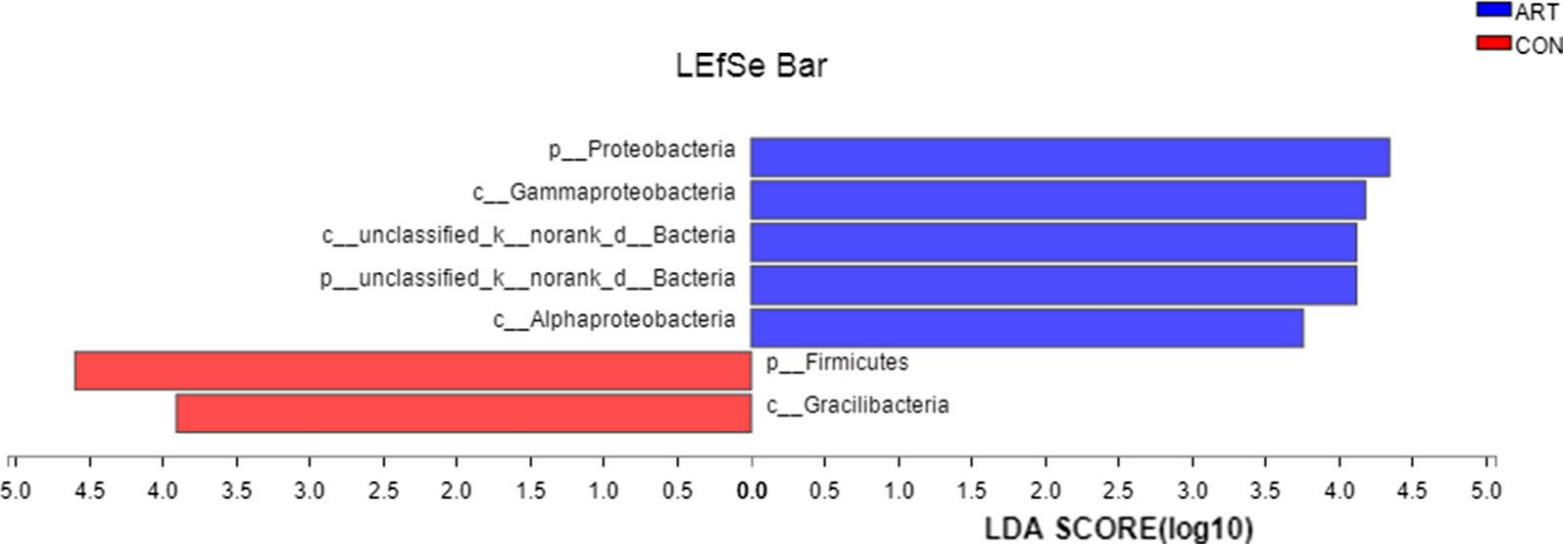
LEfSe analysis revealing significant differences in species between the ART and CON groups, with Linear discriminant analysis (LDA) scores > 3.5 and *P* value < 0.01

The LEfSe analysis revealed significant increases in *Firmicutes* and *Gracilibacteria* and reductions in *Proteobacteria*, *Gammaproteobacter*ia, *unclassified*-*k*-*norank*-*d*-*Bacteria*, *p*-*unclassified*-*k*-*norank*-*d*-*Bacteria* and *Alphaproteobacteria* in the CON group compared to the ART group (Fig. 3).

### Identification of different milk metabolites between CON vs ART

We next employed LC–MS to characterize the milk metabolome after feeding with ART. In total, 922 measurable peaks were obtained across all the milk samples. The multivariate analysis method OPLS-DA, as shown in Table 4, identified 35 significantly differential metabolites obtained from the milk samples between the ART and CON groups using VIP > 1 and *P *< 0.05. These differential metabolites are primarily glycerophospholipids, flavonoids, organooxygen compounds, fatty acyls, and glycerolipids. Milk from cows receiving ART contained higher levels of glycerophospholipids, prenol lipids, glycerolipids, flavonoids, organooxygen compounds, carboxylic acids and their derivatives and vitamins than did CON cows. In particular, the 4 glycerophospholipid metabolites phosphatidylserine (PS)(18:1(9Z)/18:1(9Z)), PS(14:1(9Z)/22:1(11Z)), PS(18:0/18:3(9Z,12Z,15Z)), and phosphatidylethanolamine (PE)(P-16:0/20:4(5Z,8Z,10E,14Z)(12OH[S])), 3 flavonoid metabolites and 2 other glycerolipid metabolites (MG(0:0/14:0/0:0) and 1-monopamitin) were elevated. In addition, carboxylic acids and their derivatives and vitamins were present at higher levels in the ART group than in the CON group.

**Table 4:** Identification of significant differential metabolites in milk from lactating cows fed TMR supplemented with 0 (CON) and 120 g/d artemisinin (ART), n = 6

Metabolites	RT (min)	Ion (m/z)	Mass error (ppm)	VIP^a^	FC^b^	*P* value	Tendency
Glycerophospholipids							
PC(18:0/20:4(5Z,8Z,11Z,14Z))	11.09	854.59	0.85	3.69	0.55	0.00	
PC(16:0/20:4(5Z,8Z,11Z,14Z))	10.62	826.56	1.16	5.75	0.70	0.01	
PC(16:0/22:5(4Z,7Z,10Z,13Z,16Z))	10.59	852.58	0.71	4.56	0.75	0.01	
LysoPC(18:0)	8.81	568.36	− 1.76	5.10	0.69	0.03	
PS(20:5(5Z,8Z,11Z,14Z,17Z)/18:2(9Z,12Z))	10.46	850.49	0.79	1.01	0.76	0.01	
PS(18:1(9Z)/18:1(9Z))	11.34	786.53	0.41	12.54	1.23	0.01	↑
PS(18:1(9Z)/22:6(4Z,7Z,10Z,13Z,16Z,19Z))	11.15	878.52	2.88	1.15	0.71	0.00	
PS(18:2(9Z,12Z)/22:6(4Z,7Z,10Z,13Z,16Z,19Z))	10.98	866.47	− 7.84	1.31	0.72	0.00	
PS(18:2(9Z,12Z)/18:0)	10.90	808.51	0.90	1.69	0.92	0.05	
1-Stearoylglycerophosphoinositol	8.50	599.32	− 0.76	8.73	0.68	0.01	
PG(16:0/0:0)	7.98	483.27	− 2.21	1.04	0.56	0.04	
PS(14:1(9Z)/22:1(11Z))	11.46	788.54	− 1.06	9.17	1.28	0.01	↑
PS(18:0/18:3(9Z,12Z,15Z))	10.96	786.53	− 1.30	4.74	1.24	0.05	↑
PE(P-16:0/20:4(5Z,8Z,10E,14Z)(12OH[S]))	10.52	740.52	− 0.51	5.81	1.22	0.01	↑
PS(18:0/18:2(9Z,12Z))	7.03	752.51	− 9.59	2.53	0.18	0.03	
Fatty acyls							
Kojibiose	0.59	377.08	0.24	17.75	0.86	0.03	
DL-2-hydroxy stearic acid	9.25	299.26	− 2.06	1.49	0.73	0.00	
2-Methylbutyroylcarnitine	2.25	246.17	0.10	5.41	0.64	0.03	
Prenol lipids							
Spirolide E	7.05	708.49	7.07	2.85	0.20	0.04	
Sphingolipids							
SM(d18:1/14:0)	10.37	719.53	− 0.30	4.85	1.20	0.02	↑
Glycerolipids							
MG(0:0/14:0/0:0)	8.08	285.24	− 0.34	3.20	2.67	0.01	↑
1-Monopalmitin	8.78	331.28	0.40	2.32	1.73	0.02	↑
Imidazopyrimidines							
Adenine	12.99	136.06	− 0.28	1.45	0.61	0.00	
Flavonoids							
Isovitexin 7-(6′’’-sinapoylglucoside) 4′-glucoside	0.76	943.26	6.16	3.83	3.81	0.02	↑
2′’-(6′’-p-Coumaroylglucosyl)quercitrin	0.80	777.16	− 4.81	6.15	0.42	0.01	
6′’-p-Coumaroylprunin	0.78	601.14	8.40	4.34	2.52	0.04	↑
Kaempferol 3-(2′’-rhamnosylgalactoside) 7-rhamnoside	0.60	777.16	− 5.28	4.62	0.21	0.00	
Kaempferol 3-neohesperidoside-7-(2′’-p-coumaryllaminaribioside)	1.15	533.16	− 4.04	3.86	1.28	0.02	↑
Organooxygen compounds							
3-Glucosyl-2,3′,4,4′,6-pentahydroxybenzophenone	0.59	445.07	− 8.72	1.99	0.25	0.00	
Maltose	0.59	341.11	− 3.21	7.52	1.18	0.00	↑
N-Acetylgalactosamine	0.64	244.08	− 1.27	1.94	0.60	0.04	
Carboxylic acids and derivatives							
5-Aminopentanamide	4.15	296.21	4.66	1.46	1.82	0.01	↑
Vitamins							
Pantothenic acid	1.831	− 0.945	55.4	3.378	1.397	0.003	↑

^a^VIP and P values: All differential metabolites ART group/CON group were those with a VIP > 1 and P < 0.05

^b^FC: fold change, the ART group vs the CON group

**Fig. 4:**
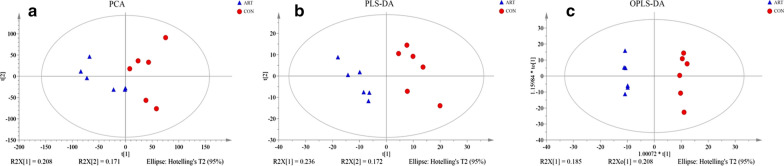
Principal component analysis (**a**), corresponding PLS-DA validation plots (**b**), and OPLS-DA score plots (**c**)

For further analysis, PCA and OPLS-DA were conducted with the CON and ART groups. As shown in Fig. 4a, axes 1 and 2 from PCA accounted for 20.8% and 17.1% of the total variation, respectively. The PLS-DA sore plots showed that the milk samples between groups were readily separable. For all the milk samples in the 95% Hotelling T^2^ ellipse, axes 1 and 2 from OPLS-DA accounted for 18.5% and 20.8% of the variation, respectively. Thus, the CON group and ART group metabolites have different compositions, indicating that PCA and OPLS-DA results reflected the difference in milk metabolites between the two groups well.

### Metabolic pathway analysis

**Fig. 5:**
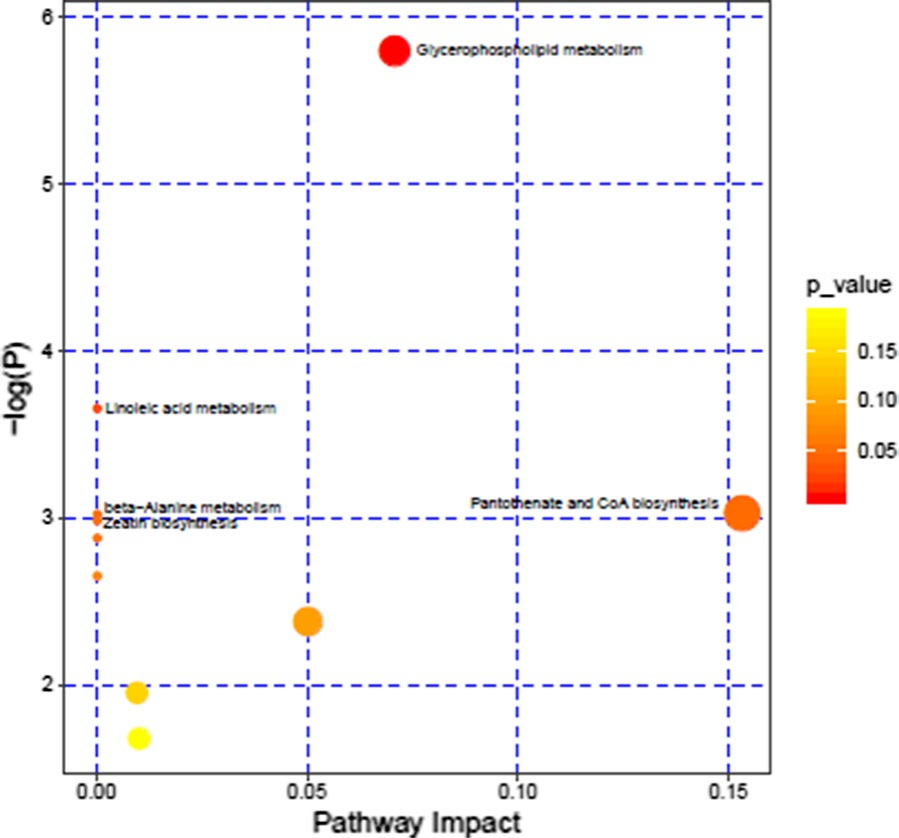
Metabolome map of differential metabolites between CON and ART dairy cows. The x-axis represents the pathway impact, and the y-axis represents the pathway enrichment. Larger symbol sizes indicate higher pathway impact values and darker colors indicate lower P values

The differential metabolites between the CON and ART groups were analyzed using KEGG pathways for functional enrichment analysis. The significantly changed pathways are shown in Table 5. Lipid metabolism, amino acid metabolism, carbohydrate metabolism, metabolism of cofactors and vitamins, metabolism of terpenoids and polyketides, and digestive system were found to be different between the two groups. Figure 5 shows that 4 metabolic pathways were enriched (*P *< 0.05): glycerophospholipid metabolism, pantothenate and CoA biosynthesis, linoleic acid metabolism and beta-alanine metabolism. Comprehensive *P* value analysis of pathways revealed that glycerophospholipid metabolism was the pathway with the greatest difference between the ART group and the CON group.

**Table 5:** Metabolic pathways and metabolites enriched in the ART group compared with the CON group

Pathway name	Class	Metabolite	*P* value
Alpha-linolenic acid metabolism	Lipid metabolism (2)	LysoPC(18:0), PC(16:0/20:4(5Z,8Z,11Z,14Z))	0.01
Linoleic acid metabolism			
Glycerophospholipid metabolism			
Lysine degradation	Amino acid metabolism (2)	5-Aminopentanamide, Pantothenic acid	0.05
Beta-alanine metabolism			
Starch and sucrose metabolism	Carbohydrate metabolism (1)	Maltose	0.05
Pantothenate and CoA biosynthesis	Metabolism of cofactors and vitamins (1)	Pantothenic acid	0.03
Zeatin biosynthesis	Metabolism of terpenoids and polyketides (1)	Adenine	0.05
Carbohydrate digestion and absorption	Digestive system (2)	Pantothenic acid; Maltose	0.05
Vitamin digestion and absorption			

In the class column, the number in () is the number of metabolites in that class

### Correlations between the milk microbiome and metabolites

**Fig. 6:**
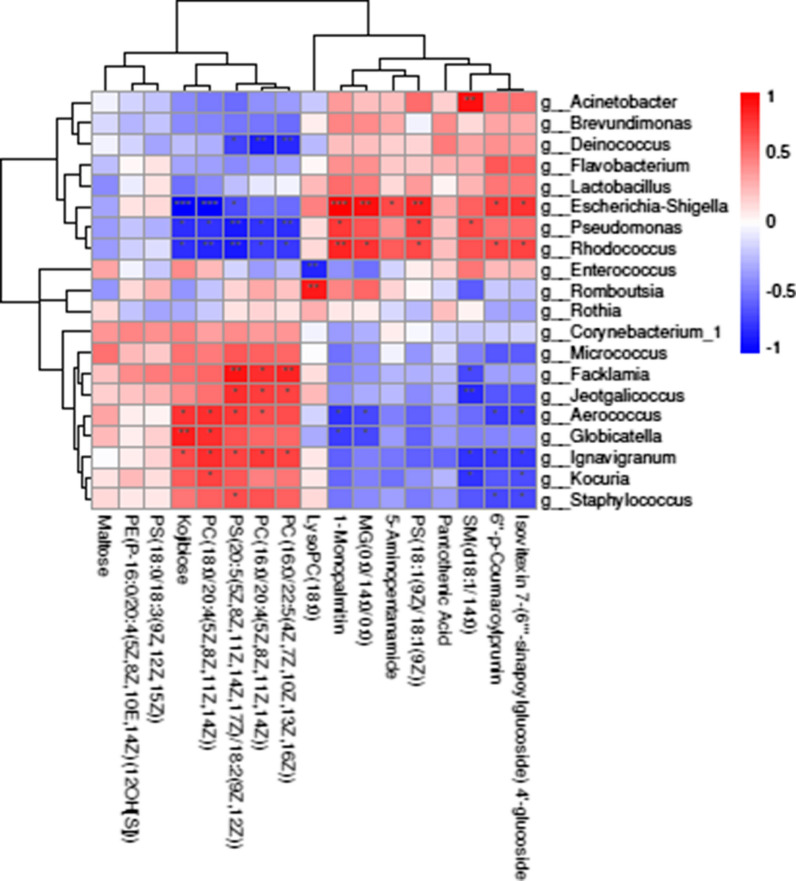
Correlation analyses between bacterial genera and metabolites in the two treatments. (*0.01 < *P *< 0.05; **0.001 < *P* ≤ 0.01; ***P ≤ 0.001)

Figure 6 shows the genus-level Spearman’s correlation coefficients for the milk microbiota and the altered metabolite profiles (r > 0.5 or <−0.5, *P *< 0.05). Figure 6 also shows that the correlations between both the presence and functions of metabolites and flora in milk are strong. We also found that *Rhodococcus, Escherichia*-*Shigella, Facklamia, Aerococcus, Staphylococcus and Ignavigranum* were remarkably correlated with the majority of metabolites (Fig. 6). Of these bacteria, *Staphylococcus, Aerococcus* and *Ignavigranum* were significantly positively correlated with PS(20:5(5Z,8Z,11Z,14Z,17Z)/18:2(9Z,12Z)) but negatively correlated with isovitexin 7-(6′’’-sinapoylglucoside) 4′-glucoside and 6′’-p-coumaroylprunin. Furthermore, the significantly decreased metabolite phosphatidylcholine (PC)(18:0/20:4(5Z,8Z,11Z,14Z)) was positively correlated with *Aerococcus, Globicatella, Ignavigranum* and *Kocuria*. The sphingomyelin (SM)(d18:1/14:0) metabolite was significantly correlated with most members of the microbiota. In addition, 1-monopalmitin was negatively correlated with *Globicatella* and *Aerococcus* but positively correlated with *Escherichia*-*Shigella, Pseudomonas* and *Rhodococcus*. Overall, we found that milk bacteria were associated with significantly altered metabolites in response to ART extract function, especially some metabolites involved in glycerolipid and flavonoid metabolism.

## Discussion

In this study, we used 16S rRNA and LC–MS metabolomics to compare milk bacteria and metabolites, revealing that ART moderately increased milk production and milk fat content and tended to decrease the SCC at the end of the treatment. Although there was no significant difference in the bacterial diversity (Shannon and Simpson indexes) between the ART and CON groups, the bacterial community richness (ACE and Chao indexes) was significantly lower in the ART group. In addition, the phylum abundances showed that *Firmicutes* was significantly decreased, while *Proteobacteria* was higher after treatment with ART. The well-recognized functional data of the milk microbiota can be used not only to identify the quality of milk but also to judge the health status of dairy cow mammary glands (Mansor 2012; Sun et al. 2017a). Correlation analysis of the microbiota and metabolites in milk revealed changes in *Aerococcus, Facklamia,* and *Staphylococcus*-related metabolites. Those identified metabolites could cause the differences in milk components between the groups. Thus, we propose that ART activities affect the organism in terms of its milk microbiota and metabolites, in turn triggering milk fat changes.

### Milk synthesis

In the present study, ART supplementation moderately increased milk yield and significantly increased milk fat rate. In line with a previous study, adding *Artemisia annua* to the cow diet increased milk production, which was attributed to the activity of phenols and flavonoids in *Artemisia annua* (Ferreira et al. 2011; Zhan et al. 2017). Furthermore, it has also been reported that plant flavonoids can increase the acetic acid concentration of dairy cows (Broudiscou et al. 2000). It is well known that acetic acid is the main precursor for milk fat synthesis; similarly, cow milk fat production can be significantly increased by intravenous acetic acid injection (Storry and Rook 1965). Therefore, milk fat increase might be caused by rumen acetate acid changes, which warrants further investigation in future studies.

In addition, the SCC tended to decrease in the ART group compared with the CON group. The SCC is one of the most useful and widely used tools to predict mammary gland health in bovines (Tong et al. 2019); thus, our results proved that ART could reduce mastitis susceptibility. Similar to the results of previous studies by Zhan et al. (Zhan et al. 2017), feeding 60 mg/kg alfalfa flavones could reduce the SCC, alter the composition of milk, improve antioxidant properties and affect immunity in dairy cows. In addition, members of the microbiota such as corynebacteria in milk are usually associated with low-SCC intramammary infections (IMIs) (Guan et al. 2014). Accordingly, the ART antimicrobial and anti-inflammatory properties function by inhibiting the synthesis of cell walls and membranes, interfering with enzymes in or on specific cells and inhibiting bacterial proliferation that causes mastitis in dairy cows (Cushnie and Lamb 2011), especially when modulating the rumen or gut microbiota (Amaretti et al. 2015; Zhan et al. 2017). Similar results were also obtained in our study; the relative abundance of *Firmicutes* was significantly decreased with ART supplementation, whereas that of *Proteobacteria* was significantly increased. These species are similar to members of the rumen microbiome (Wang et al. 2018), and we hypothesize that the bacteria can be transferred from the rumen to the mammary gland. Therefore, ART may be associated with a systemic and local immune responsiveness of the udder that is accompanied by microbiota changes.

### Multivariate analysis of milk

16S rRNA sequencing and analysis provide a low-cost and high-yield method for the evaluation of milk microflora (Caporaso et al. 2011; Hélène et al. 2016). In the present study, milk microbial richness was significantly decreased in the ART group. Recent investigations of the bovine milk microbiota show that it usually has a variety of bioactive or probiotic properties to resist the defense mechanism of the udder (F et al. 2016). Additionally, the effect of plant bacteriostatic factors on the rumen microbiota or pathogens of mastitis in dairy cows (Durmic et al. 2008; Cushnie and Lamb 2011) may also be associated with milk microbiota changes in the present study. Similarly, antimicrobials could reduce the diversity of microbial ecosystems and, in alternative stable forms, accompany this with reduced species richness (Lozupone and Knight 2006). Henderson et al. (Henderson et al. 2013) also reported that sample extraction may also affect DNA quality and impact microbiota diversity. Moreover, previous research found that milk microbial diversity was related to mastitis (Hélène et al. 2016). Therefore, no difference in microbiota diversity was caused by the ART treatment, which will strengthen the use of ART as novel prophylactic or therapeutic product (or both) alternatives to antimicrobials in dairy cows.

In our current study, *Firmicutes, Proteobacteria, Actinobacteria* and *Bacteroidetes* were the major phyla in the milk of the two groups, and the predominant genera were *Aerococcus, Corynebacterium_1, Staphylococcus* and *Enterococcus,* which is consistent with previous studies (Zhang et al. 2017). Similar results were also obtained in the current study; *Firmicutes* was significantly decreased with ART supplementation, whereas *Probacteria* was significantly increased. These species are similar to those in the rumen microbiome (Jinjin et al. 2018; Wang et al. 2018). Furthermore, recent reports support this entero-mammary pathway via lymphatic and peripheral blood circulation in humans and mice (Rodríguez, 2014) via transfer from mothers to neonates by the gut-breast axis (Jost et al. 2014).

It has been shown that mastitis in cows or goats by *Staphylococcus* (Guan et al. 2014) causes serious losses in dairy products and to the animal husbandry industry (Tong et al. 2019). Increases in the relative abundances of *Corynebacterium boris*, *Aerococcus* and *Staphylococcus* increase the SCC in the milk of dairy cows (Hogan et al. 1988; Sun et al. 2017b). Furthermore, the contents of *Corynebacterium, Facklamia* and *Aerococcus* were higher in dairy cows with a history of clinical mastitis than in healthy dairy cows (Hooman et al. 2018). These pathogens can be found in healthy dairy cows because they are an important cause of mastitis when the proportions of bacteria are out of balance (Guan et al. 2014). In our study, the *Staphylococcus, Corynebacterium* and *Aerococcus* abundances were extremely reduced in the milk from the ART group; these genera were the most commonly identified genera omnipresent in the dairy environment and gained great attention as the leading bacteria in IMI (Pyrl and Taponen 2009; Vanderhaeghen et al. 2014; Hooman et al. 2018). Our study also detected that *Pseudomonas* was negatively correlated with most metabolites associated with the ART treatment. (Figure 6). It has been reported that *Pseudomonas* causes milk deterioration by producing lipase and proteolytic enzymes, and the quality of milk can be maintained by controlling its growth (Chikage et al. 2018). When the lipase content increased, the hydrolysis of triglycerides in dairy cows and long-chain fatty acid production increased. These mechanistic insights into the microbiota response to ART may have important implications for understanding how the milk microbiota participates in biosynthesis regulation and in relation to udder health and mastitis.

### Differences in milk metabolites

Metabolomics is an emerging area of research involving organisms; its methods detect small molecular metabolites in samples and enable a comprehensive understanding of biological processes (Sun et al. 2017a). We used LC–MS to analyze the milk metabolite response to ART supplementation. In total, 35 different metabolites were identified between the ART and CON groups. Some metabolites were upregulated, such as glycerolipids (MG(0:0/14:0/0:0)), flavonoids (isovitexin 7-(6’’’-sinapoylglucoside)4′-glucoside and 6′’-p-coumaroylprunin), carboxylic acids and their derivatives (5-aminopentanamide), and vitamins (pantothenic acid). MG is a monoacylglyceride product of triglycerides that regulates liver development and activity (Coleman and Haynes, 1984). In our study, MG was significantly increased (by 2.67-fold) in the ART group, which strengthens the idea that ART could trigger the mammary gland response to milk biosynthesis, and a remarkably negative correlation was found between MG and *Aerococcus*. Meanwhile, MG is absorbed more easily than other fatty acid derivatives in the intestine; this absorption increases the content of docosahexaenoic acid and increases the antioxidant activity of mouse plasma (Cho et al. 2009). A plausible hypothesis, similar to one previously reported, is that the mechanisms underlying the effects of nutrition on the potential involvement of the microbiota in the microbiome-gut-brain axis (Cryan and O’Mahony, 2011) are worthy of further study.

In the present study, we found that the flavonoids increased the most of all the metabolites, followed by isovitexin 7-(6′’’-sinapoylglucoside)4′-glucoside (by 3.81-fold) and 6′’-p-coumaroylprunin (by 2.52-fold), in the ART group. These metabolites were negatively correlated with *Staphylococcus*. Recent studies have shown that flavonoids have antibacterial and antioxidant properties that promote the production and quality of animal products (Zhan et al. 2017). Furthermore, flavonoids increase the activity of antioxidant enzymes to enhance antioxidant capacity and protect tissues and cells from free radical-mediated damage (Xiao-Shuang et al. 2015). These findings may partially explain the moderate SCC decrease induced by ART supplementation. Interestingly, we detected that many metabolites were significantly correlated with members of the microbiota that were potential pathogens, such as *Staphylococcus, Facklamia,* and *Aerococcus*. Our findings collectively highlighted the observed microbiota and metabolic changes and provided further insight into the performance of specific ART-related functions in milk profiles that can be used to analyze the differences in milk synthesis between the groups. We speculated that this discrepancy might be associated with the decrease in the SCC resulting from ART supplementation.

PS and PE are important components of biomembrane structure (Cole et al. 2012). The phospholipids in milk are mainly PE, PC and SM (Shoji et al. 2006). PC, which plays a key role in lipid metabolism, can synthesize very low density lipoprotein (VLDL) and be used for the export of triacylglycerol (TAG) in the liver (Cole et al. 2012). Furthermore, aggregation of ATG in the liver may increase fatty liver production (Elke et al. 2016). The current study revealed that PC and PS production was downregulated and PE production was upregulated in the ART group compared with the CON group. Furthermore, the mammary gland utilizes blood PC as a cellular energy source for the production of glycerophosphocholine and free fatty acids and can synthesize milk triglycerides and phospholipids (Easter et al. 1971). Thus, this might explain the mechanism by which the milk fat content was significantly increased in the ART group; specifically, we revealed that glycerophospholipid metabolites were significantly reduced by ART treatment. However, the content of PC was lower in the CON group because when the PC breakdown rate decreased, which is closely associated with negative energy balance (NEB) and fat mobilization, causing metabolic disorders and ketosis (Klein et al. 2012). In conclusion, our results indicate that the glycerol phospholipid metabolic pathway may be the main component of the mechanism by which ART affects milk quality.

### Key differential metabolic pathways between the two groups

Based on a comprehensive analysis of important metabolic pathways that identified 33 differential milk metabolites between the two groups, lipid metabolism may be the most important pathway for milk quality improvement induced by ART. An unavoidable NEB accompanies various metabolic disorders and affects the immune system, and the energy and lipid metabolism of dairy cows is abnormal and vigorous, causing many diseases (Bouvier-Muller et al. 2018). However, the conversion of PC into milk fat could protect free blood fatty acids, thus reducing the mobilization of body fat (Klein et al. 2012). It is reasonable to conclude that ART protects the health of the body. Furthermore, ART supplementation significantly increased β-alanine metabolism relative to that of the control group. β-Alanine metabolism mainly occurs in muscle and brain, and its final metabolite is acetic acid (Griffith 1986). Meanwhile, β-alanine can improve the antioxidant capacity of the body(Smith et al. 2012), which may be the reason for the improvement in milk quality. In addition, pantothenate and CoA biosynthesis was also significantly upregulated by ART. Pantothenic acid is a water-soluble vitamin. When it is transformed into CoA or bound to acyl carrier protein (ACP) in vivo, it mainly participates in the metabolism of fatty acids, carbohydrates and energy and significantly reduces the levels of cholesterol and triglycerides (Smith and Song 1996). This may reduce abnormalities in lipid and energy metabolism in dairy cows. Therefore, our data indicate that glycerophospholipids and glycerolipids could be potential biomarkers in the milk response to ART feed in dairy cows, which further supports the functional link between ART and milk changes. Taken together, the results in our study support the assumption that ART changes substances in milk by maintaining lipid metabolism in the mammary gland.

In summary, LC–MS and 16S rRNA was used to analyze milk metabolomics and bacterial community profiles for dairy cows, revealing that ART supplementation increased milk fat, decreased the SCC and may affect the structures of bacterial communities, metabolites and metabolic pathways. Moreover, ART decreased the relative abundances of *Corynebacterium_1*, *Aerococcus*, *Staphylococcus* and *Facklamia*. Our results also revealed that some of the 33 metabolites that changed significantly in milk after ART supplementation were potential biomarkers that respond to ART. In addition, glycerophospholipids and glycerolipids could be potential biomarkers in the milk response to ART feed in dairy cows, and ART changes substances in milk by protecting lipid metabolism in the mammary gland. This study has provided further insights into the mechanisms at the metabolic level that can improve understanding of the effects of ART addition. Overall, our findings provide new strategies for improving milk quality with the use of ART; however, they warrant further investigations for identification of potential immune regulation mechanisms underlying the effects of ART on dairy cows.

## Supplementary information

**Additional file 1.** Company qualification and artemisinin analysis certificate.

## Data Availability

All raw sequences were submitted to the NCBI Sequence Read Archive (SRA: http://www.ncbi.nlm.nih.gov/Traces/sra/) under accession number SRP 254006. The data supporting the conclusion of this article are included in this article.
