# Supplementary material for: Microbiome and metabolic changes in milk in response to artemisinin supplementation in dairy cows
Source: AMB Express. 2020 Aug 24;10:154. doi: 10.1186/s13568-020-01080-w (PMC7445214; doi:10.1186/s13568-020-01080-w)
Supplement: Supplementary file 1 — Additional file 1. Company qualification and artemisinin analysis certificate. [file 13568_2020_1080_MOESM1_ESM.docx]

**Additional file**

the *Artemisia annua* extract used in this experiment was purchased from Shanxi Senfu Co., Ltd. (Shanxi, China) and company did the analysis. We upload this company qualification (Supplement 1) and the certificate of analysis (Supplement 2).


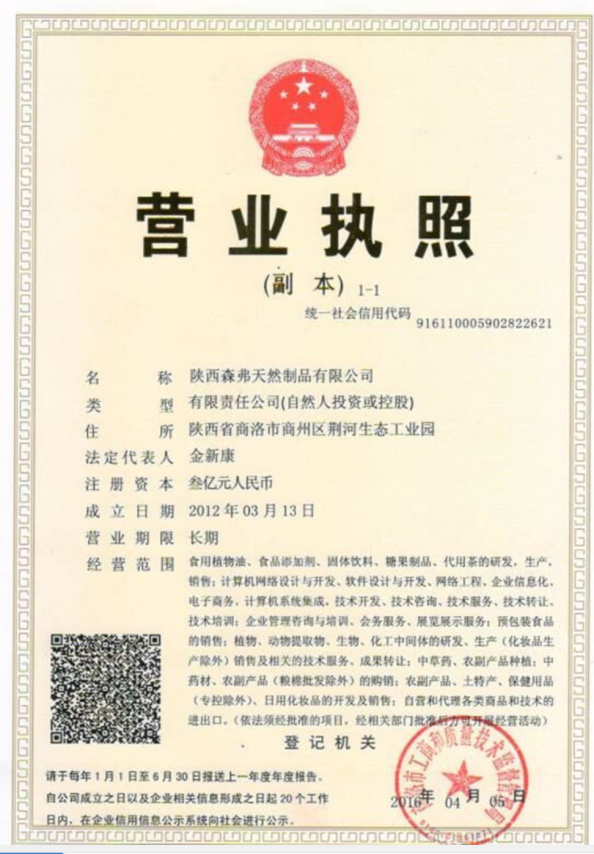


Fig.1 company qualification


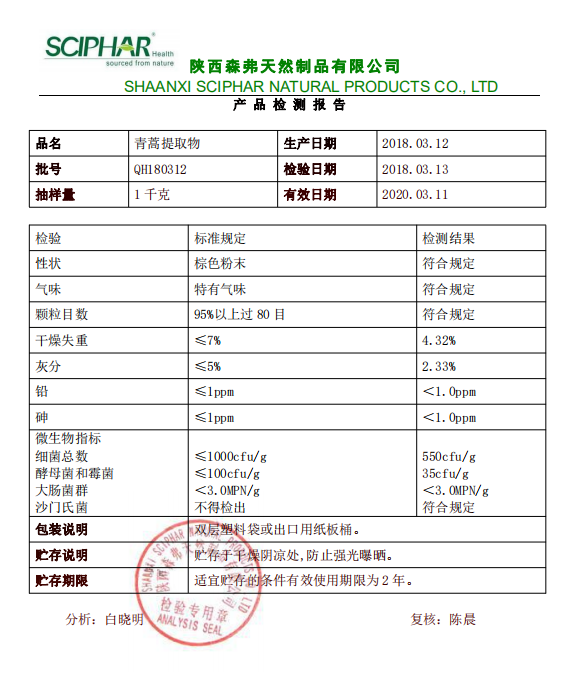


Fig.2 the certificate of analysis
